# A Duplex Quantitative Real-Time Reverse Transcription-PCR for Simultaneous Detection and Differentiation of Flaviviruses of the Japanese Encephalitis and Ntaya Serocomplexes in Birds

**DOI:** 10.3389/fvets.2020.00203

**Published:** 2020-04-21

**Authors:** Maia Elizalde, Cristina Cano-Gómez, Francisco Llorente, Elisa Pérez-Ramírez, Laia Casades-Martí, Pilar Aguilera-Sepúlveda, Francisco Ruiz-Fons, Miguel Ángel Jiménez-Clavero, Jovita Fernández-Pinero

**Affiliations:** ^1^Centro de Investigación en Sanidad Animal (CISA), Instituto Nacional de Investigación y Tecnología Agraria y Alimentaria (INIA), Valdeolmos-Alalpardo, Spain; ^2^Instituto de Investigación de Recursos Cinegéticos IREC (CSIC-UCLM-JCCM), SaBio Group, Ciudad Real, Spain; ^3^Epidemiología y Salud Pública, CIBERESP, Madrid, Spain

**Keywords:** flavivirus, Japanese encephalitis serocomplex, Ntaya serocomplex, West Nile virus, diagnostics, duplex real-time RT-PCR

## Abstract

High impact, mosquito-borne flaviviruses such as West Nile virus (WNV), Usutu virus (USUV), Japanese encephalitis virus (JEV), Tembusu virus (TMUV), and Bagaza/Israel turkey meningoencephalomyelitis virus (BAGV/ITV) are emerging in different areas of the world. These viruses belong to the Japanese encephalitis (JE) serocomplex (JEV, WNV, and USUV) and the Ntaya serocomplex (TMUV and BAGV/ITV). Notably, they share transmission route (mosquito bite) and reservoir host type (wild birds), and some of them co-circulate in the same areas, infecting overlapping mosquito and avian population. This may simplify epidemiological surveillance, since it allows the detection of different infections targeting the same population, but also represents a challenge, as the diagnostic tools applied need to detect the whole range of flaviviruses surveyed, and correctly differentiate between these closely related pathogens. To this aim, a duplex real-time RT-PCR (dRRT-PCR) method has been developed for the simultaneous and differential detection of JE and Ntaya flavivirus serocomplexes. The method has been standardized and evaluated by analyzing a panel of 49 flaviviral and non-flaviviral isolates, and clinical samples of different bird species obtained from experimental infections or from the field, proving its value for virus detection in apparently healthy or suspicious animals. This new dRRT-PCR technique is a reliable, specific and highly sensitive tool for rapid detection and differentiation of JE and Ntaya flavivirus groups in either domestic or wild animals. This novel method can be implemented in animal virology diagnostic laboratories as screening tool in routine surveillance and in the event of bird encephalitis emergence.

## Introduction

The genus Flavivirus, within the family *Flaviviridae*, comprises more than 70 different viruses, many of which represent relevant pathogens for humans and animals (1, 2). Serological affinities allowed their classification into eight antigenic groups or serocomplexes (3). Two of these, namely Japanese encephalitis (JE) and Ntaya groups, comprise a number of pathogenic viruses associated with neurological diseases in different vertebrate species, including domestic species and human beings, being most of them potentially or factually zoonotic (4–7). Clinically relevant viruses within the JE group are, for instance, West Nile virus (WNV), Saint Louis encephalitis virus (SLEV), Japanese encephalitis virus (JEV), Murray Valley encephalitis virus (MVEV), and Usutu virus (USUV), while the Ntaya serocomplex includes Bagaza virus (BAGV), its synonymous Turkey meningoencephalomyelitis virus (ITV) (8), and Tembusu virus (TMUV). All these viruses are maintained in nature in a cycle involving avian reservoir hosts and *Culex* spp. mosquitoes. Also, all of them have caused an increasing number of outbreaks over the last years (9–11). In fact, the incidence and geographic spread of these flaviviral infections has risen dramatically worldwide and should be regarded as a threat to animal and human health (12). In Europe and the Mediterranean region, increasing flavivirus activity has been observed in recent times (13). The number of WNV outbreaks has intensely risen since late 1990's (14–16) and USUV has spread widely since its first detection in Austria in 2001 (17–19). In 2010, BAGV emerged in Southern Spain (20) in an area where WNV and USUV were co-circulating in the same avian population (21). Its synonymous virus, ITV, also re-emerged in Israel in the same time period (22). Likewise, in other areas of the world, similar patterns of flavivirus emergence are being observed, particularly involving those belonging to the JE and Ntaya groups (7, 23, 24). Also, the risk of emergence of any of those viruses in distant territories should not be disregarded, as some members of these groups have demonstrated their capacity to undergo transcontinental displacements. Notably, WNV was able to reach the Americas in 1999, probably introduced from the Mediterranean area (25). Similarly, USUV and BAGV were able to reach Europe likely from Sub-Saharan Africa (12, 20, 26).

As the number of flaviviruses circulating in given geographic areas (such as those already mentioned) grows, molecular diagnosis of flaviviral infections relies more and more on generic RT-PCR approaches, which may be particularly advantageous in bird disease diagnostics and surveillance. However, pan-flavivirus PCR methods described so far are focused essentially on public health application or entomological surveillance (27–33), and no PCR-based system is currently available for avian monitoring. Most important bird-pathogenic flaviviruses belong to the above-mentioned JE and Ntaya serocomplexes. The generic detection of viral species of both serocomplexes in a single test would potentially provide more accurate and rapid diagnostic results in monitoring programs, where high-sensitive methods are demanded for large screening. This study describes the development and standardization of a quantitative duplex real-time RT-PCR (dRRT-PCR) method for the simultaneous detection and differentiation of flaviviruses from the JE and Ntaya serocomplexes, to be used as a screening tool in routine avian surveillance and in the event of bird encephalitis outbreaks.

## Materials and Methods

### Viruses

A collection of 49 different viral isolates was used for the development and standardization of the dRRT-PCR assay (Table 1). Briefly, a flavivirus panel composed of 27 isolates from JE serocomplex, 7 isolates from Ntaya serocomplex and 5 reference strains of other flavivirus species was employed. When needed, viral isolates were propagated and titrated by cell culture standard techniques. All flavivirus isolates used in the study belong to the virus collection held in reserve at INIA-CISA, Valdeolmos, Spain, and were originally obtained from different providers or collaborators as described in Table 1.

**Table 1:** Flavivirus isolates used in this study and results obtained by the dRRT-PCR and the RT-PCR methods used as reference.

**Flavivirus sero complex**	**Virus**	**Isolate (lineage)**	**Geographical origin**	**Species of origin**	**Year of isolation**	**Source**	**Duplex RRT-PCR**		**Reference PCR**
							**Fam (JE)**	**Joe (Ntaya)**	
Japanese encephalitis (JE)	WNV	E-101 (1a)	Egypt	Human	1951	Institut Pasteur Lyon (France)	21.33	No Ct	22.8^a^
		IS-98 STD (1a)	Israel	White stork	1998	ANSES (France)	29.55	No Ct	31.2^a^
		NY99 034EDV (1a)	New York	American crow	1999	Diagnostic Virology Laboratory USDA Ames (USA)	17.34	No Ct	17.02^a^
		04.05 (1a)	Morocco	Horse	2003	Biopharma Rabat (Morocco)	18.26	No Ct	19.57^a^
		GE-1b/B (1a)	Spain	Golden eagle	2007	INIA-CISA Madrid (Spain)	18	No Ct	19.73^a^
		15803 (1a)	Italy	Magpie	2008	IZSLER Brescia (Italy)	23.48	No Ct	26.61^a^
		225677/2009 (1a)	Italy	Jay	2009	IZSLER Brescia (Italy)	24.55	No Ct	25.46^a^
		233184/4/2009 (1a)	Italy	Black headed gull	2009	IZSLER Brescia (Italy)	24.19	No Ct	26.87^a^
		Spain/2010/H-1b (1a)	Spain	Horse	2010	INIA-CISA Madrid (Spain)	20.74	No Ct	20.98^a^
		Spain/2012 (1a)	Spain	Horse	2012	LCV Algete (Spain)	19.35	No Ct	20.57^a^
		Kunjin KJ359-11 (1b)	Australia	Horse	1984	ISCIII Madrid (Spain)	29.4	No Ct	33.67^a^
		B956 (2)	Uganda	Human	1937	ATCC (USA)	16.53	No Ct	17.07^a^
		Austria/2008 (2)	Austria	Goshawk	2008	VetMedUni Vienna (Austria)	26.48	No Ct	31.8^a^
		WN/Horse/RSA/1/08 (2)	South Africa	Horse	2008	Onderstepoort Veterinary Institute Pretoria (South Africa)	31.48	No Ct	35.22^a^
		178907/2013 (2)	Italy	*Culex pipiens*	2013	IZSLER Brescia (Italy)	16.72	No Ct	19.32^a^
		Rabensburg 97-103 (3)	Czech Republic	*Culex pipiens*	1997	ISCIII Madrid (Spain)	18.78	No Ct	Pos^c^
		MP502-66 (putative 6)	Malaysia	*Culex pseudovishnui*	1966	ISCIII Madrid (Spain)	25.86	No Ct	Pos^c^
		Koutango ArD96655 (putative 7)	Senegal	*Rhipicephalus guihoni*	1993	Institute Pasteur Dakar (Senegal)	16.42	No Ct	Pos^c^
	USUV	SAAR 1776/1958	South Africa	*Culex neavei*	1958	ANSES (France)	24.77	No Ct	28.31^a^
		939	Austria	Blackbird	2001	ISCIII Madrid (Spain)	19.9	No Ct	22.25^a^
		231247/2011	Italy	Blackbird	2011	IZSLER Brescia (Italy)	22.19	No Ct	25.37^a^
		206795-3/2012	Italy	*Culex pipiens*	2012	IZSLER Brescia (Italy)	24.77	No Ct	28.31^a^
		229615-1/2013	Italy	*Culex pipiens*	2013	IZSLER Brescia (Italy)	23.3	No Ct	28.11^a^
		HautRhin7315/France/2015	France	Blackbird	2015	ANSES (France)	25.39	No Ct	28.27^a^
	JEV	Nakayama	Japan	–	1935	ISCIII Madrid (Spain)	23.9	No Ct	Pos^c^
	MVEV	MV/1/1951	–	–	1951	ISCIII Madrid (Spain)	26.91	No Ct	Pos^c^
	SLEV	FL52	–	–	–	ISCIII Madrid (Spain)	38	No Ct	Neg^c^
Ntaya	BAGV	Spain H/2010	Spain	Red-legged partridge	2010	INIA-CISA Madrid (Spain)	No Ct	25.46	24.71^b^
	ITV	Vaccine strain	Israel	Domestic turkey	1985	Kimron Veterinary Institute (Israel)	No Ct	27.73	26.74^b^
		618	Israel	Domestic turkey	1995	Kimron Veterinary Institute (Israel)	No Ct	26.34	24.53^b^
		107458	Israel	Domestic turkey	2010	Kimron Veterinary Institute (Israel)	No Ct	19.19	17.61^b^
		106819	Israel	Domestic turkey	2010	Kimron Veterinary Institute (Israel)	No Ct	29.11	27.68^b^
		105520	Israel	Domestic turkey	2010	Kimron Veterinary Institute (Israel)	No Ct	30.69	27.96^b^
	TMUV	UVE/TMUV/1955/MY/MM1775	Malaysia	*Culex tritaeniorhinchus*	1955	European Virus Archive (EVAg)	No Ct	19.85	Pos^c^
Other flaviviruses	TBEV	Neudorfl	–	–	–	ISCIII Madrid (Spain)	No Ct	No Ct	Pos^c^
		Hypr	–	–	–	ANSES (France)	No Ct	No Ct	Pos^c^
	LIV	–	–	–	–	ISCIII Madrid (Spain)	No Ct	No Ct	Pos^c^
	ZIKV	–	–	–	–	ISCIII Madrid (Spain)	No Ct	No Ct	Pos^c^
	DENV	Hawaii	–	–	–	ISCIII Madrid (Spain)	No Ct	No Ct	Pos^c^

TBEV, Tick-borne encephalitis virus; LIV, Louping-ill virus; ZIKV, Zika virus; DENV, Dengue virus.

a Del Amo et al. (34).

b Buitrago et al. (35).

c *Scaramozzino et al. (27)*.

Additionally, a set of 10 avian or equine non-flavivirus isolates were analyzed in the specificity studies, namely: Avian influenza virus (AIV, subtypes H5N2, H7N1, H1N1), Newcastle disease virus (NDV), African horse sickness virus (AHSV, serotype 4), Western equine encephalitis virus (WEEV), Equine herpesvirus-1 (EHV-1), Venezuelan equine encephalitis virus (VEEV), Equine influenza virus (EIV, subtype H3N8), and Vesicular stomatitis virus (VSV, Indiana serotype). AIV isolates were provided by IZSLER (Brescia, Italy); WEEV, EHV-1 and VEEV were obtained from ANSES (Maisons-Alfort, France); NDV, AHSV, EIV, and VSV belong to the virus collection maintained at INIA-CISA (Valdeolmos, Spain).

#### Experimental and Field Samples

Clinical samples collected from *in vivo* experiments carried out with different bird species (house sparrow, red-legged partridge and gray partridge) in the BSL-3 animal facilities at INIA-CISA (36–38) were used for this particular study. Specifically, a panel of 20 immature feathers, 20 blood samples and 24 tissues (heart, liver, brain, spleen, and kidney) obtained from non-infected control birds, and 2 blood, 2 immature feathers and 20 tissue samples collected at different times post-infection from birds experimentally inoculated with WNV or BAGV were analyzed (Table 2A).

**TABLE 2A:** Clinical samples used in this study and results obtained by the dRRT-PCR and the RT-PCR methods used as reference; experimental samples from *in vivo* experiments performed at INIA-CISA.

**Virus**	**Species**	**Animal Id**	**Type of Sample**	DPI^*^	**DUPLEX RRT-PCR**		**WNV-1/WNV-2/USUV RRT-PCR**^**a**^			**BAGV RRT-PCR^**b**^ (Ct)**
					**JE (Ct)**	**NTAYA (Ct)**	**WNV-1 (Ct)**	**WNV-2 (Ct)**	**USUV (Ct)**	
WNV L1 (GE-1b/B Spain 2007)	House sparrow	11	Brain	7	28.44	No Ct	31.25	No Ct	No Ct	
			Kidney		27.37	No Ct	29.15	No Ct	No Ct	
			Spleen		24.01	No Ct	25.68	No Ct	No Ct	
			Heart		26.85	No Ct	28.53	No Ct	No Ct	
			Liver		24.78	No Ct	27.15	No Ct	No Ct	
WNV L2 (Austria/2008)	Red-legged partridge	7	Brain	7	28.41	No Ct	No Ct	32.45	No Ct	
			Kidney		26.43	No Ct	No Ct	31.20	No Ct	
			Spleen		27.6	No Ct	No Ct	35.19	No Ct	
			Heart		24.48	No Ct	No Ct	30.89	No Ct	
			Liver		36.75	No Ct	No Ct	39.03	No Ct	
			Blood		35.84	No Ct	No Ct	No Ct	No Ct	
			Feather		23.38	No Ct	No Ct	30.01	No Ct	
BAGV (Spain H/2010)	Red-legged partridge	4	Brain	6	No Ct	31.19				29.54
			Kidney		No Ct	26.17				25.81
			Spleen		No Ct	24.31				25.69
			Heart		No Ct	23.47				25.81
			Liver		No Ct	26.05				26.84
	Grey partridge	4	Brain	4	No Ct	35.83				27.11
			Kidney		No Ct	22.97				21.19
			Spleen		No Ct	24.27				19.66
			Heart		No Ct	29.16				20.5
			Liver		No Ct	26.37				22.45
			Blood		No Ct	24.95				20.81
			Feather		No Ct	23.28				19.98

* *DPI: days-post-infection. ^a^Del Amo et al. (34). ^b^Buitrago et al. (35)*.

On the other hand, a set of 9 WNV-positive (lineage 1) field samples (5 feathers, 2 swabs, and 2 brain samples) collected from different avian species were included in this study. The samples were obtained from WNV cases occurred in wild birds in Southern Spain, and were provided as WNV PCR positive by the National Reference Laboratory (NRL) for avian diseases, Laboratorio Central de Veterinaria (LCV, Algete, Spain). Seven samples (feather and tissues) obtained from a red-legged partridge found dead during the BAGV outbreaks occurred in Southern Spain in 2010, which was submitted by Estación Biológica de Doñana (EBD-CSIC, Seville, Spain) for diagnostic confirmation, were incorporated to this study (Table 2B).

**TABLE 2B:** Clinical samples used in this study and results obtained by the dRRT-PCR and the RT-PCR methods used as reference; field samples from wild birds collected in Spain.

**Virus**	**Species**	**Source/** **Animal Id**	**Type of Sample**	**DUPLEX RRT-PCR**		**WNV-1/WNV-2/USUV RRT-PCR**^**a**^			**BAGV RRT-PCR^**b**^ (Ct)**
				**JE (Ct)**	**NTAYA (Ct)**	**WNV-1 (Ct)**	**WNV-2 (Ct)**	**USUV (Ct)**	
WNV L1	Red-legged partridge	LCV/30/11	Brain	28.54	No Ct	31.36	No Ct	No Ct	
		LCV/2496/15	Cloacal swab	38.10	No Ct	33.71	No Ct	No Ct	
		LCV/3179/13	Oral swab	31.60	No Ct	34.83	No Ct	No Ct	
	White stork	LCV/2439/16-1	Feather	36.89	No Ct	No Ct	No Ct	No Ct	
	Black vulture	LCV/2442/16-5	Brain	24.34	No Ct	28.17	No Ct	No Ct	
		LCV/2442/16-4	Feather	28.28	No Ct	30.75	No Ct	No Ct	
	Cinereous vulture	LCV/2442/16-3	Feather	31.66	No Ct	31.49	No Ct	No Ct	
	Duck	LCV/2439/16-11	Feather	27.58	No Ct	30.43	No Ct	No Ct	
		LCV/2858/16	Feather	34.10	No Ct	39.15	No Ct	No Ct	
	Little owl	UEX/10B	Feather	22.34	No Ct	26.36	No Ct	No Ct	
			Brain	24.65	No Ct	25.69	No Ct	No Ct	
			Liver	24.67	No Ct	27.64	No Ct	No Ct	
			Lung	18.79	No Ct	21.54	No Ct	No Ct	
			Heart	23.21	No Ct	26.09	No Ct	No Ct	
			Spleen	34.38	No Ct	38.32	No Ct	No Ct	
		UEX/A	Feather	19.38	No Ct	22.26	No Ct	No Ct	
			Brain	27.62	No Ct	29.25	No Ct	No Ct	
			Liver	25.26	No Ct	28.48	No Ct	No Ct	
			Lung	18.85	No Ct	21.89	No Ct	No Ct	
			Heart	19.70	No Ct	22.96	No Ct	No Ct	
			Spleen	26.77	No Ct	30.64	No Ct	No Ct	
			Kidney	24.21	No Ct	25.51	No Ct	No Ct	
USUV	Common blackbird	IREC/A18/177	Oral swab	35.99	No Ct	No Ct	No Ct	39.37	
			Cloacal swab	No Ct	No Ct	No Ct	No Ct	No Ct	
BAGV	Red-legged partridge	EBD/2010A	Feather	No Ct	24.98				23.26
			Brain	No Ct	28.98				28.62
			Kidney	No Ct	28.86				28.3
			Eye	No Ct	22.72				22.47
			Heart	No Ct	32.93				33.13
			Muscle	No Ct	32				32.08
			Skin	No Ct	29.98				28.34

Only the results of the individuals giving positive result to any JE or Ntaya flavivirus serogroup are shown.

a Del Amo et al. (34).

b *Buitrago et al. (35)*.

In addition, a panel of 25 feathers and tissue samples (heart, liver, brain, lung, spleen and kidney), obtained from 4 individuals (2 little owls, 1 goshawk, and 1 crow) showing neurological disorders were also incorporated in the study. These samples remained undiagnosed and were submitted by the Veterinary Faculty of Universidad de Extremadura (UEX, Cáceres, Spain) for diagnostic investigation (Table 2B).

Finally, the dRRT-PCR technique was transferred to IREC (Ciudad Real, Spain) and applied in a surveillance study of wild birds in Castilla-La Mancha (Spain). Briefly, a total of 237 birds were sampled, belonging to the following families: *Alcedinidae* (*n* = 1), *Certhiidae* (*n* = 6), *Corvidae* (*n* = 20), *Emberizidae* (*n* = 15), *Fringillidae* (*n* = 10), *Hirundinidae* (*n* = 13), *Laniidae* (*n* = 8), *Muscicapidae* (*n* = 1), *Oriolidae* (*n* = 1), *Paridae* (*n* = 61), *Passeridae* (*n* = 36), *Prunellidae* (*n* = 1), *Sylviidae* (*n* = 27), and *Turdidae* (*n* = 36). As far as possible, blood, immature feathers and oral and cloacal swabs were collected; the feather being the sample tested, when available, in the PCR screening.

#### Nucleic Acid Extraction

Total RNA was extracted at INIA-CISA (Valdeolmos, Spain) from 200 μl of sample (virus suspension, blood, feathers, swabs and tissue homogenates 10% in PBS) using the automated BioSprint 15 workstation and the BioSprint DNA blood kit (Qiagen, Valencia, CA), according to manufacturer's instructions with small modifications (carrier RNA was added to AL buffer for a final concentration of 5 μg/ml). Finally, RNA was recovered in 100 μl of nuclease-free water.

The Macherey-Nagel NucleoSpin TriPrep kit (Fisher Scientific, Leicestershire, UK) was used for RNA extraction of field samples at IREC (Ciudad Real, Spain), following the protocol recommended by the manufacturer, using 200 μl of sample and recovering RNA in 50 μl of nuclease-free water.

#### Duplex Real-Time RT-PCR: Design and Methodology

A comprehensive selection of Ntaya and JE serocomplex viruses' full-length genome sequences available from GenBank was individually aligned using Clustal Omega software (European Bioinformatic Institute, Hinxton, Cambridge CB10 1SD, UK). Two primers and TaqMan probe set, each specific for one serogroup covering all representative virus species were designed, targeting non-structural protein 2A (NS2A) gene and 3' end non-coding region (3'NCR) for JE and Ntaya groups, respectively. Properties of the designed primers and probes were analyzed *in silico* with Primer Express 2.0 (Applied Biosystems, Life Technologies Corporation, Carlsbad, CA, USA). Probes were labeled with different reporter dyes, FAM for JE serocomplex and JOE for Ntaya serocomplex, to allow a simultaneous, but differential detection of both groups in a single run (Table 3). A BLASTn search of the selected primers and probes sequences against the GenBank database confirmed the specificity to the corresponding JE or Ntaya flavivirus serogroup.

**TABLE 3:** List of primers and probes designed for the detection of JE and Ntaya serocomplexes by dRRT-PCR assay.

**Primer/Probe name**	**Sequence and labeling (5' → 3')^**a**^**	**Nucleotide positions**	**Product size**	**Target genome region**
**Japanese encephalitis serocomplex**
JE-3533F	TCAGYTGGGCCTTCTGGT	3,533–3,550^b^	65 bp	NS2A
JE-3597R	TGGCCGTCCACCTCTTSC	3,597–3,580^b^		
JE-FAM-3553	6FAM-TGTTYTTGGCCACCCAGGAGGT -BHQ1	3,553–3,574^b^		
**Ntaya Serocomplex**
NT-10510F	GTTGGATGACGGTGCWGYCTG	10,510–10,530^c^	171 bp	3′ NTR
NT-10680R	GCTGCACTGCATGCTARTGGC	10,680–10,660^c^		
NT-JOE-10594	JOE-TACCGWCTCGGAGARCTCCCTGGC-BHQ1	10,594–10,617^c^		

Primers and probes were purchased from Roche Applied Science (Spain).

a Code for mixed bases positions: R:A/G, Y:C/T, S:G/C, and W:A/T.

b Nucleotide positions regarding to the WNV L1 Spain/2010/H-1b strain. GenBank accession no. JF719069.

c *Nucleotide positions regarding to the BAGV Spain H/2010 strain. GenBank accession no. HQ644143*.

Initially, RRT-PCR assays were optimized individually for JE or Ntaya serocomplex detection under the same reaction conditions, to be afterwards modified into a duplex format using the commercial AgPath-ID one-step RT-PCR kit (Life Technologies, Thermo Fisher Scientific, USA). For the dRRT-PCR, a reaction mix was prepared containing (per vial) 3 μl of RNA template, 0.75 μM of each primer and 0.2 μM of each probe (Table 3), 0.8 μl of 25X RT-PCR enzyme mix (containing ArrayScript™ Reverse Transcriptase and AmpliTaq Gold® DNA Polymerase) and 10 μl of 2X RT-PCR Buffer (includes ROX™ passive reference dye for quantitative fluorescent signal normalization), and nuclease-free water to reach a reaction volume of 20 μl. Reaction mixes for individual RRT-PCR assays were prepared with same reagents, volumes and concentrations described for the dRRT-PCR, except only primers/probe for the target flaviviral serocomplex were added. All reactions were carried out using Mx3005P equipment and software (Stratagene Inc., La Jolla, CA, USA) using the following thermal profile: reverse transcription at 48°C for 20 min, initial PCR activation step at 95°C for 10 min, followed by 45 cycles of 15 s at 95°C and 1 min at 60°C. The fluorescence signal emitted by FAM and JOE reporter dyes was measured simultaneously and independently at the end of each cycle. A threshold cycle value (Ct) >40 was set as negative result.

#### Construction of *in vitro*-Transcribed RNA Standards

Two ssRNA standard controls were produced for analytical sensitivity estimation of the dRRT-PCR. Specifically, WNV-L1 GE-1b/B and BAGV Spain H/2010 strains were used as templates in a PCR using outer primers of the duplex assay. The resulting products were sequenced to ensure the specificity of the amplification. Likewise, the cDNAs were gel-purified with Wizard SV Gel and PCR Clean-Up System (Promega, USA) and quantified by spectrophotometry. Each cDNA was cloned into pGEM-T easy vector system (Promega, USA) and DNA plasmids were further purified using Wizard Plus SV Miniprep DNA purification system (Promega, USA) and finally quantified by spectrophotometry. *In vitro* transcription was performed with Riboprobe® *in vitro* Transcription System (Promega, USA) over linearized DNA plasmid, following the manufacturer's instructions. RNA transcripts were then purified using MEGAclear Transcription Clean-Up Kit (Ambion, Thermo Fisher Scientific, USA), treated with 2U of Turbo™ DNase (Ambion, Thermo Fisher Scientific, USA) and re-purified with TriPure Isolation Reagent (Roche Applied Science, Germany). The produced RNA standards were quantified by ND-1000 NanoDrop spectrophotometer (Thermo Fisher Scientific, USA). Mean concentration values were 1.57 E^+11^ and 4.72 E^+11^ RNA copies/μl for WNV-L1 and BAGV *in vitro*-transcribed RNA control preparations, respectively. A series of dilutions were generated for each quantified WNV and BAGV RNA and stored as standard RNA samples for further use in sensitivity assays.

Best-fit lines (standard curves) were calculated by the least squares regression method from the Ct values obtained for the serial dilutions of the two RNA standards produced to determine the dynamic range and detection limit of the dRRT-PCR method.

#### Reference RT-PCR Methods

Two previously validated techniques, namely a triplex RRT-PCR for WNV (lineages 1 and 2) and USUV simultaneous detection (34) and a single RRT-PCR for BAGV detection (35), were employed as reference methods in comparative assays. For detection of other flaviviruses, a widely used conventional RT-PCR (27) was performed with minor modifications, and amplification products were further sequenced to confirm the virus identity.

## Results

### Analytical Performance: Dynamic Range, Linearity, Efficiency, and Detection Limit

Initially, the analytical sensitivity of the dRRT-PCR was studied by analyzing duplicates of 10-fold serial dilutions of viral suspensions of WNV-L1 (Spain/2010/H-1b), WNV-L2 (B956), USUV (SAAR 1776/1958), and BAGV (Spain H/2010). All experiments were run in parallel with the equivalent reference RRT-PCR method (34, 35), obtaining a similar (BAGV) or at least 10 times higher (WNV-L1, WNV-L2, USUV) sensitivity with the dRRT-PCR (Supplementary Table 1).

**Figure 1:**
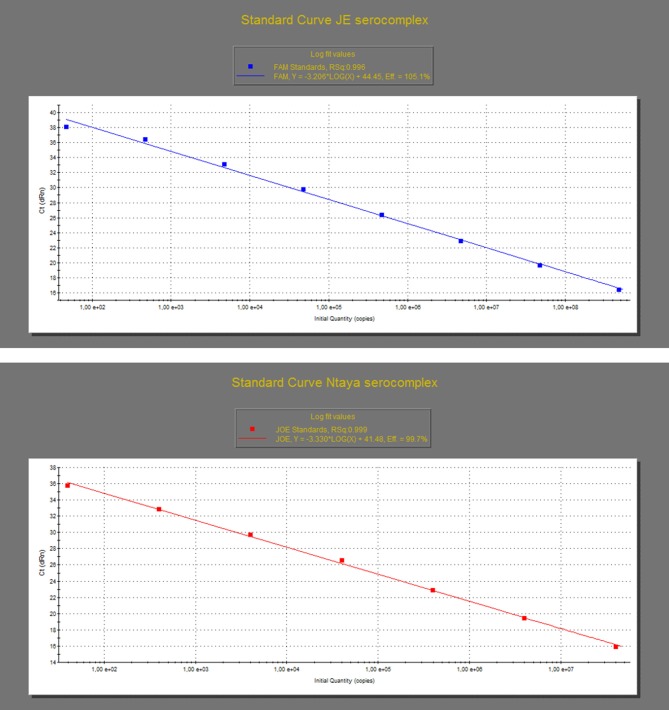
Standard curves of the dRRT-PCR for the detection of JE (top graph) and Ntaya flavivirus serocomplexes (bottom graph). The curves were generated by analysis of triplicates of 10-fold serial dilutions of the quantified *in vitro*-transcribed WNV (top graph) and BAGV (bottom graph) RNA standards produced as synthetic positive controls. Each ▪ corresponds to the mean value of three replicates.

The analytical performance of the dRRT-PCR method was assessed by analyzing the *in vitro-*transcribed RNA standards produced for WNV and BAGV, as above described. Specifically, the dilutions containing a range from 1.57 E^+8^ to 1.57 E^+0^ RNA copies/μl for WNV-L1 and 1.32 E^+7^ to 1.32 E^+0^ RNA copies/μl for BAGV were tested in triplicates to construct the standard curves for both JE and Ntaya serocomplexes, respectively. The assays reacted in a dose-dependent manner with each reference RNA standard along a dynamic range of eight and seven 10-fold dilutions for both WNV-L1 and BAGV, respectively. The detection limit of the dRRT-PCR was estimated to be below 50 RNA copies for both JE and Ntaya serogroups. The standard curve produced with the WNV-L1 RNA standard showed a value of correlation coefficient (R) of 0.996 and efficiency rate (E) of 105.1%. For BAGV RNA standard, R was 0.999 and the efficiency of the dRRT-PCR was 99.7% (Figure 1, Supplementary Table 2).

Finally, the capacity of the dRRT-PCR to detect the presence of two target viruses in a single sample was evaluated with a set of WNV+BAGV RNA standards combinations. Specifically, dilutions of both RNA standards representing strong (Ct <20) and weak (Ct > 33) positive samples were mixed in equivalent and disparate proportions. The panel of artificial “co-infected samples” were analyzed in triplicates and in parallel with triplicates of the corresponding “single infected samples.” All preparations were correctly scored and Ct values were similar for each dilution analyzed pooled or individually (Supplementary Table 3).

### Specificity Assays and Detection Range

To assess the analytical specificity of the dRRT-PCR, a panel of RNAs from 39 different flavivirus isolates and 10 avian and equine non-flavivirus isolates were analyzed. Fluorescence signals were obtained correctly for the range of JE and Ntaya serocomplex isolates analyzed, being able to clearly differentiate between them without any cross-reactions. The reference RRT-PCR techniques were carried out in parallel for comparison, the dRRT-PCR reporting similar or lower Ct values for the range of WNV-L1, WNV-L2, and USUV isolates tested, while some slightly higher Ct values were obtained for BAGV/ITV isolates (Table 1). The specificity of the assay was further proved since no fluorescence signal was reported with any heterologous flavivirus (Table 1) and non-flavivirus isolates (data not shown).

### Performance With Clinical Samples

The performance of the developed dRRT-PCR method for practical use in diagnosis was assessed initially by analyzing a panel of samples (*n* = 24), including blood, feathers, heart, spleen, liver, kidney, and brain, obtained from house sparrows, red-legged partridges and gray partridges experimentally infected with GE-1b/B Spain 2007 strain of WNV-L1, Austria/2008 strain of WNV-L2, or Spain H/2010 strain of BAGV, at INIA-CISA BSL-3 animal facilities (36–38). All experimental samples were correctly reported by the duplex assay, overall showing similar or lower Ct values for those samples infected with WNV-L1 or WNV-L2 than those obtained using the corresponding reference technique. For samples infected with BAGV, Ct values scored by the dRRT-PCR were generally higher than by the reference RRT-PCR (Table 2A). No fluorescence signal was obtained when experimental samples (*n* = 64) from non-infected control birds were tested, confirming the diagnostic specificity of the dRRT-PCR (data not shown).

A first collection of WNV-L1 positive field samples (*n* = 9) provided by the Spanish NRL was subjected to analysis to evaluate the diagnostic sensitivity of the dRRT-PCR. All feathers, swabs and tissues were scored correctly in the JE serocomplex. Running in parallel the reference RRT-PCR, most WNV-L1 samples reported lower Ct values in the dRRT-PCR, and even one feather was missed by the triplex reference method (Table 2B). In addition, all samples (*n* = 7) of a red-legged partridge (*Alectoris rufa*) found dead during the outbreaks of BAGV in 2010 were reported as positive for the Ntaya serocomplex by the dRRT-PCR, and were confirmed as BAGV positive by the reference technique. Similar Ct values were obtained for most samples by the two techniques (Table 2B).

To evaluate the competence of the dRRT-PCR with undiagnosed field material, a panel of feathers and tissue samples (*n* = 25) of different wild birds (2 little owls, 1 goshawk, and 1 crow), that had died with neurological signs, were examined in parallel by the dRRT-PCR and the two reference RRT-PCR techniques. An infection due to JE serocomplex virus was detected in all samples of the two little owls *(Athene noctua)* by the dRRT-PCR and WNV-L1 was identified by the triplex reference RRT-PCR, reporting the dRRT-PCR lower Ct values in all positive samples (Table 2B). Samples from the other two individuals remained negative by all assays.

Finally, the developed dRRT-PCR technique was implemented as screening tool in a surveillance study carried out in Castilla-La Mancha (Spain) by IREC. Of 237 sampled wild birds, 175 feathers, 58 oral swabs, and 4 cloacal swabs were analyzed. One feather from a common blackbird (*Turdus merula*) was found positive (Ct = 15) for the JE serocomplex. Further sequencing confirmed the infection due to USUV in this bird. Unfortunately, this sample could not be analyzed by the triplex reference RRT-PCR. Alternatively, oral and cloacal swabs of the same blackbird were examined at INIA-CISA by the dRRT-PCR and the WNV-L1/WNV-L2/USUV triplex reference RRT-PCR, obtaining a weak positive signal in the oral swab for JE serocomplex and USUV, respectively (Table 2B).

### Repeatability Assessment

Positive extraction controls employed throughout this study were used to assess the intra- and inter-assay repeatability of the dRRT-PCR. These were prepared diluting two viral suspensions of WNV-L1 (as positive control for JE serocomplex) and BAGV (as positive control for Ntaya serocomplex) until getting the dilutions to give an expected Ct value of 30 ± 2 for each target. Aliquots of the two WNV and BAGV positive controls were stored at −20°C and further included in each RNA extraction run.

The analysis of 10 WNV and 10 BAGV positive extraction controls in 10 duplex RRT-PCR runs proved the inter-assay repeatability, obtaining mean Ct values of 30.39 and 30.04, with a standard deviation (SD) of 0.59 and 0.43, respectively. Finally, RNAs (stored at −20°C) from the same batch of WNV and BAGV positive extraction controls (*n* = 10+10) were tested in one single dRRT-PCR run to evaluate the intra-assay repeatability, giving a mean Ct value of 30.84 (SD = 0.74) for WNV and of 30.57 (SD = 0.59) for BAGV.

## Discussion

The recent emergence of different flaviviruses in wide regions of the world makes co-circulation of these pathogens in same geographic areas more likely (12, 13, 39). Furthermore, there is a range of flavivirus species sharing vectors and hosts, producing similar disease pattern in susceptible animals. This is of current relevance for the Japanese encephalitis (JE) and Ntaya serogroups, which include *Culex*-borne viral species infecting the same bird population, in which they can produce a similar encephalitic disease. The increasing spread and incidence of the viruses belonging to these two groups requires that avian surveillance plans, in countries where they can potentially emerge and circulate, implement methods capable of detecting any of them. These methods could be of particular interest in certain geographic regions such as Southern Spain, Israel and several African countries (e.g., South Africa or Senegal) where WNV, USUV, and BAGV/ITV are present and infect bird and mosquito population (21, 40–42).

Although some pan-flavivirus PCR methods have been described so far, most are focused on public health application or entomological surveillance (27–33), while none of them has been developed for birds. This is significant in the current epidemiological situation, where the recent and potential emergence of bird-pathogenic flaviviruses in different territories poses a complex challenge for the diagnostic laboratories and for the veterinary authorities. On the other hand, the combination of the relevant bird-pathogenic flaviviruses species (those belonging to the JE and Ntaya serocomplexes) in a single assay makes very difficult to develop a PCR test with the high sensitivity level demanded for a screening tool. This was solved, in this study, by designing a duplex RRT-PCR (dRRT-PCR) method for the generic and differential detection of these two target flavivirus serogroups. It is well-known that the design of generic molecular tools may be challenging to cover the range of target pathogens and may limit their sensitivity (43), especially when clinical material is analyzed. However, the new dRRT-PCR has demonstrated to be a specific and highly sensitive tool, capable of detecting the wide range of JE and Ntaya flaviviral species analyzed, and posing a performance similar to the RRT-PCR methods used as reference (34, 35). On the other hand, most PCR methods described for flaviviruses detection have been verified with human or mosquito samples, when available (28, 31–33). In this study, WNV, USUV and BAGV positive samples from a variety of wild bird species were available for the evaluation of the dRRT-PCR. The results analyzing an extensive panel of clinical material from experimental studies and from the field have proved its diagnostic capacity. In this regards, it deserves to point out the usefulness showed by the dRRT-PCR in the detection of viral infection in undiagnosed field samples (WNV in little owls) and in outbreak investigations (BAGV in red-legged partridge). Furthermore, the implementation of the dRRT-PCR technique in a wild bird monitoring study allowed the identification of a common blackbird infected with a JE-serogroup virus that was further confirmed as USUV. This is a relevant finding to support the potential value of this method for its use as a screening tool in routine diagnosis. Therefore, by combining two methods, one for JE and one for Ntaya in one duplex assay it is possible to detect a wide range of bird flaviviruses, including most important ones. In addition, by differentiating these two groups in one single analysis, we obtain a useful information to narrow the range of suspicious agents to analyse in a second, more specific method.

This quantitative method can also be used to estimate viral loads in blood and/or other organs or samples. Albeit not mandatory for diagnostic or surveillance studies, this ability can be used to monitor the clinical course of the infection and to determine if a given species develops enough viremia to act as a competent host (able to transmit the virus to the mosquito vector), which is essential to understand its role in the epidemiology of these flaviviruses.

Finally, the new dRRT-PCR was developed bearing in mind the current situation in some Mediterranean countries, where different flaviviruses of the two target serogroups have emerged and co-circulate in the avian population. However, this method can also be very useful in other geographic regions such as South-East Asia, where viruses belonging to the Ntaya serocomplex, e.g., Tembusu and Tembusu-related viruses, are spreading into areas where Japanese encephalitis virus is historically present (23, 44–47). As well, according to the epidemiological scenario, the RRT-PCR can be turned into individual tests for the only detection of JE or Ntaya serogroup providing equal diagnostic performance than the duplex format.

In conclusion, the duplex quantitative real-time RT-PCR described in this study provides a novel tool for the diagnostic and epidemiological surveillance of JE and Ntaya serocomplex flaviviruses, comprising a wide range of arboviral pathogens threatening animal and public health worldwide. This new method allows for the rapid detection and differentiation of these two serocomplexes being especially helpful as screening tool in bird flavivirus surveillance and in the diagnosis of avian encephalitis cases.

## Data Availability Statement

All datasets generated for this study are included in the article/Supplementary Material.

## Ethics Statement

The animal study was reviewed and approved by INIA animal experimentation ethics committee and by the authorized organ of the Department of Environment and Land Management of the Community of Madrid.

## Author Contributions

JF-P conceived and designed the study, analyzed the data, and amended the article. ME participated in the design and development of the method and supervised the technical work. CC-G contributed to the design and development of the method and in the laboratory tests. CC-G, FL, and EP-R participated in sample selection and analysis and contributed to draft the article. PA-S contributed to sample selection and analysis. FR-F and LC-M collected and analyzed most of the field samples. MJ-C participated in the study design, coordination, and drafted the article. All authors read and approved the final manuscript.

## Conflict of Interest

The authors declare that the research was conducted in the absence of any commercial or financial relationships that could be construed as a potential conflict of interest.
