# Supplementary material for: A Duplex Quantitative Real-Time Reverse Transcription-PCR for Simultaneous Detection and Differentiation of Flaviviruses of the Japanese Encephalitis and Ntaya Serocomplexes in Birds
Source: Front Vet Sci. 2020 Apr 21;7:203. doi: 10.3389/fvets.2020.00203 (PMC7186316; doi:10.3389/fvets.2020.00203)
Supplement: Supplementary file 1 [file Data_Sheet_1.PDF]

Supplementary Material

Supplementary Table 1. Analytical sensitivity of the dRRT-PCR by analysis of duplicates of 10-fold serial dilutions of viral suspensions of WNV-L1 (Spain/2010/H-1b), WNV-L2 (B956), USUV (SAAR 1776/1958) and BAGV (Spain H/2010). Results obtained in parallel with the equivalent reference RRT-PCR method are shown. Mean Ct values and standard deviation (SD) obtained are shown. [1] Del Amo et al. 2013. [2] Buitrago et al. 2012

Virus	Titre ($TCID_{50}$/ml)	Dilution	Duplex RRT-PCR		WNV-1/WNV-2/USUV RRT-PCR[1]			BAGV RRT-PCR[2] Ct (SD)
			JE Ct (SD)	NTAYA Ct (SD)	WNV-1 Ct (SD)	WNV-2 Ct (SD)	USUV Ct (SD)	
WNV L1 (Spain/2010/H-1b)	1,26E+6 TCID50/ml	1,00E-01	14.25 (0.08)	No Ct	17.81 (0.02)	No Ct	No Ct	No Ct
		1,00E-02	17.44 (0.26)	No Ct	21.58 (0.49)	No Ct	No Ct	No Ct
		1,00E-03	20.33 (0.19)	No Ct	26.06 (0.11)	No Ct	No Ct	No Ct
		1,00E-04	24.34 (0.23)	No Ct	30.34 (0.17)	No Ct	No Ct	No Ct
		1,00E-05	26.72 (0.56)	No Ct	33.20 (0.32)	No Ct	No Ct	No Ct
		1,00E-06	31.53 (0.16)	No Ct	37.59 (0.85)	No Ct	No Ct	No Ct
		1,00E-07	33.37 (0.72)	No Ct	No Ct	No Ct	No Ct	No Ct
		1,00E-08	35.22 (0.00)	No Ct	No Ct	No Ct	No Ct	No Ct
		1,00E-09	No Ct	No Ct	No Ct	No Ct	No Ct	No Ct
WNV L2 (B956)	5,24E+5 TCID50/ml	1,00E-01	20.24 (0.61)	No Ct	No Ct	22.25 (0.14)	No Ct	No Ct
		1,00E-02	24.03 (0.34)	No Ct	No Ct	25.68 (0.00)	No Ct	No Ct
		1,00E-03	27.89 (0.49)	No Ct	No Ct	29.66 (0.13)	No Ct	No Ct
		1,00E-04	30.67 (0.23)	No Ct	No Ct	33.22 (0.08)	No Ct	No Ct
		1,00E-05	33.22 (0.18)	No Ct	No Ct	36.50 (0.02)	No Ct	No Ct
		1,00E-06	36.16 (0.37)	No Ct	No Ct	No Ct	No Ct	No Ct
		1,00E-07	No Ct	No Ct	No Ct	No Ct	No Ct	No Ct
USUV (SAAR 1776/1958)	1,76E+8 TCID50/ml	1,00E-01	16.09 (0.40)	No Ct	No Ct	No Ct	17.67 (0.13)	No Ct
		1,00E-02	18.93 (0.30)	No Ct	No Ct	No Ct	22.23 (0.16)	No Ct
		1,00E-03	22.82 (0.03)	No Ct	No Ct	No Ct	26.84 (0.49)	No Ct
		1,00E-04	26.97 (0.36)	No Ct	No Ct	No Ct	30.52 (0.02)	No Ct
		1,00E-05	30.71 (0.00)	No Ct	No Ct	No Ct	35.07 (0.13)	No Ct
		1,00E-06	33.65 (0.47)	No Ct	No Ct	No Ct	38.49 (0.32)	No Ct
		1,00E-07	35.89 (0.97)	No Ct	No Ct	No Ct	No Ct	No Ct
		1,00E-08	No Ct	No Ct	No Ct	No Ct	No Ct	No Ct
BAGV (Spain H/2010)	6,34E+5 TCID50/ml	1,00E-01	No Ct	19.82 (0.18)	No Ct	No Ct	No Ct	18.68 (0.09)
		1,00E-02	No Ct	22.86 (0.37)	No Ct	No Ct	No Ct	21.89 (0.13)
		1,00E-03	No Ct	26.91 (0.17)	No Ct	No Ct	No Ct	26.40 (0.06)
		1,00E-04	No Ct	30.04 (0.81)	No Ct	No Ct	No Ct	29.71 (0.04)
		1,00E-05	No Ct	33.90 (0.39)	No Ct	No Ct	No Ct	33.72 (0.32)
		1,00E-06	No Ct	36.24 (0.34)	No Ct	No Ct	No Ct	36.13 (0.56)
		1,00E-07	No Ct	No Ct	No Ct	No Ct	No Ct	No Ct

Supplementary Table 2. Analytical sensitivity of the dRRT-PCR by analysis of triplicates of 10-fold serial dilutions of the quantified in vitro-transcribed WNV (JE serocomplex representative) and BAGV (Ntaya serocomplex representative) RNA standards produced as synthetic positive controls. Mean Ct values and standard deviation (SD) obtained are shown.

Virus	Standard RNA copies	Duplex RRT-PCR	
		JE Ct (SD)	NTAYA Ct (SD)
WNV	4,71E+08	16.45 (0.38)	No Ct
	4,71E+07	19.68 (0.23)	No Ct
	4,71E+06	22.92 (0.08)	No Ct
	4,71E+05	26.39 (0.11)	No Ct
	4,71E+04	29.78 (0.03)	No Ct
	4,71E+03	33.13 (0.20)	No Ct
	4,71E+02	36.54 (1.04)	No Ct
	4,71E+01	38.17 (0.98)	No Ct
BAGV	3,96E+07	No Ct	15.95 (0.09)
	3,96E+06	No Ct	19.41 (0.25)
	3,96E+05	No Ct	22.90 (0.46)
	3,96E+04	No Ct	26.54 (0.14)
	3,96E+03	No Ct	29.72 (0.13)
	3,96E+02	No Ct	32.85 (0.27)
	3,96E+01	No Ct	35.80 (0.31)

Supplementary Table 3. Analytical performance study of the dRRT-PCR by analysis of triplicates of single and pooled dilutions of the two in vitro-transcribed RNA standards produced as synthetic positive controls. Mean Ct values and standard deviation (SD) obtained are shown.

Virus	RNA standard/s dilution	Duplex RRT-PCR	
		JE Ct (SD)	NTAYA Ct (SD)
WNV	1,00E-04	18.57 (SD 0.19)	No Ct
	1,00E-08	34.26 (SD 0.21)	No Ct
WNV+BAGV	1E-4 + 1E-5	19.99 (SD 0.23)	20.40 (SD 0.28)
	1E-4 + 1E-9	19.87 (SD 0.34)	35.80 (SD 0.99)
	1E-8 + 1E-5	34.10 (SD 0.21)	20.00 (SD 0.15)
	1E-8 + 1E-9	34.33 (SD 0.13)	34.06 (SD 0.50)
BAGV	1,00E-05	No Ct	18.97 (SD 0.12)
	1,00E-09	No Ct	34.25 (SD 1.00)
